# Irisin interaction with adipose tissue secretions by exercise training and flaxseed oil supplement

**DOI:** 10.1186/s12944-019-0960-4

**Published:** 2019-01-17

**Authors:** Hossein Shirvani, Saleh Rahmati-Ahmadabad

**Affiliations:** 1 Exercise Physiology Research Center, Life Style Institute, Baqiyatallah University of Medical Sciences, Nosrati alley, Sheikh Bahaei Street, Mollasadra Street, Vanak Square, Post Office Box: 19395-5487, Tehran, Iran; 2 Department of Physical Education, Pardis branch, Islamic Azad University, Pardis, Iran

**Keywords:** Adipokines, Flaxseed oil supplement, High intensity interval Tarining (HIIT), Metabolic crosstalk, Myokines

## Abstract

**Background:**

Previous studies have shown that physical training and natural diet able to change the expression and concentration of peptides and proteins. Myokines and adipokines play an important role in metabolism and metabolic syndrome. Therefore, the purpose of the present study was to investigate the effect of high-intensity interval training (HIIT) and supplementation of flaxseed oil on plasma irisin, nesfatin-1 and resistin in male rats.

**Methods:**

Forty adult male rats were randomly divided into four groups (ten in each group) including Control-Saline (CS), Training-Saline (TS), Control-FlaxOil supplement (CO), and Training-FlaxOil supplement (TO). The training groups performed for 10 weeks and 5 sessions each week, interval training with 90–95% VO2max on rodent treadmill, and supplement groups received flaxseed oil (300 mg / kg). Five days after the last training session, rats were sacrificed. Blood samples were taken from the heart and plasma was evaluated.

**Results:**

Exercise Training significantly increased plasma levels of irisin (*P* = 0.019), nesfatin-1 (*P* = 0.01), and decreased resistin (*P* = 0.01). Flaxseed oil significantly reduced plasma resistin levels (*P* = 0.02). Plasma irisin levels in the supplementation group were higher than all groups (*P* = 0.041).

**Conclusion:**

There was a significant positive correlation between plasma levels of irisin with nesfatin-1 and negative correlation with resistin. HIIT program with flaxseed oil as a modality can create a metabolic crosstalk between skeletal muscle and adipose tissues and have health benefits.

## Background

Adipose and skeletal muscle tissues produce various cytokines and their interaction can potentially alter cell metabolism [1, 2]. Irisin is a remarkable myokine/adipokine/neurokinin that has recently attracted the attention of researchers [3]. Irisin secretion improves metabolic syndrome and glycemia [4]. Skeletal muscle irisin induces muscle hypertrophy [5], and also acts on other tissues such as adipose tissues and induces weight loss by converting white fat into brown fat [6]. The mechanism of the effects of irisin on adipose tissue is has not been clearly understood. A previous study [7] showed that irisin affects white adipocytes via browning genes including Uncoupling Protein 1 (UCP1), Cell Death-Inducing DFFA-Like Effector A (CIDEA), CarnitinePalmitoyltransferase 1B (CPT1B), and Deiodinase, Iodothyronine Type 2 (DIO2). In the present study, we suggest and thus examine the effect of irisin on adipose tissue via changes in energy balance peptide such as nesfatin and resistin. Nesfatin-1 and resistin are important adipokines that act like irisin in affecting weight loss, insulin resistance and metabolic syndrome [8]. Thus, the first aim of this study was the evaluation of the correlation between muscle irisin with adipose tissue nesfatin-1 and resistin.

Previous studies have shown that physical training and the use of natural substances can affect the gene expression and plasma levels of proteins, peptides, and hormones [9, 10]. With regard to physical activity, studies have shown that different modes of physical activity can alter the expression of tissue/plasma irisin [11], nesfatin-1 [12] and resistin [13]. In recent times, high-intensity interval training (HIIT) is a novel and enjoyable type of exercise training [14]. It has been reported that HIIT affects exercise capacity, endocrine hormones, and fat mobilization due to its lower time requirement compared to other types of training [15]. There is a dearth in researches on the effect of HIIT on irisin, nesfatin-1, and resistin. Thus, this study also aims at evaluating the effects of HIIT on irisin, nesfatin-1, and resistin. With regard to natural substances, flaxseed is rich in omega-3 (ω-3) fatty acid, α-linolenic acid (ALA) and phytoestrogen lignans [16]. Fibers and ligands of flaxseed have been reported to have beneficial health effects such as weight loss and reduced metabolic disorders [17, 18]. To the best of the author’s knowledge, there have been no research on the effects of flaxseed on irisin, nesfatin-1, and resistin, hence, this study also examined this effect. In summary, the combination of exercise with natural substances may have a varied effect than exercise or natural substances alone. Overall, the aim of the present study was to investigate the effects of HIIT with and without flaxseed oil on plasma irisin, nesfatin-1, and resistin concentration, in addition to their interaction on plasma irisin, nesfatin-1 and resistin.

## Methods

### Animals

Forty adult male Wistar rats were randomly selected for the present study.

### Ethical considerations

The present study was conducted with the written consent of the research deputy of Baqiyatallah University. This study was conducted in accordance with National Institutes of Health (NIH) publication and all ethical conventions regarding laboratory animals were considered.

### Animal conditions

Animals were kept in the animal houses of Baqiyatallah University of Medical Science at a temperature of 22 ± 2degrees, humidity 45–50% and lighting-dark cycle (12 h light, 12 h darkness) in special cages and their palms were covered with clean wood chips. Special compressed food produced by the Behparvar Manufacturing Company in Karaj and urban filtered water in 500 ml bottles, were used for laboratory rats.

### Animal groups

Rats were divided randomly into four groups (ten per group) including Control-Saline (CS), Training-Saline (TS), Control-FlaxOil supplement (CO), and Training-FlaxOil supplement (TO).

### Flaxseed oil

Fresh flaxseeds were collected from growing areas in the city of Mehriz, Yazd Province. The oil was extracted by oil-making machine after approval from the Faculty of Biology (Department of Botany) in Baqiyatallah University and was administered to the rats in the relevant groups based on their weight at a dose of 300 mg/kg. Saline was administered to the other groups. The supplement and saline were administered using oral gavage before the exercise.

### Orientation to HIIT

The orientations of rats were performed with high periodic intense training protocol of 10 practice sessions in 2 weeks. This indicated that on the first day of training, the rats were placed on a treadmill with all precision and comfort. Moreover, it began to practice with a low and even speed. In the next sessions, the rats were joined to the plans in order to be familiar with the periodic protocol that was used with low speeds of periodic exercise. During the 2 weeks, exercise time was increased until the rats reached the real time of main body exercise in 18 min.

### Main HIIT protocol

After 2 weeks, the main exercise began and was completed for 10 weeks. It should be noted that the slope of the treadmill during the whole exercise training was 0° and familiarity was conducted for the non-exercise groups. At the end of 2 weeks familiarity, VO2 max was estimated in rats. According to the exercise protocol, the first main session began based on the percentage of estimated VO2 max described as m/min [19].

Due to lack of direct access to tools such as the respiratory gas analyzing device, the exact method based on a study by Hoydal et al., [19] was performed to describe VO2max as m/min. This protocol was carefully used as follows: first, warm-up was performed at low speed (10 m/min) for 10 min. Afterwarming up, test were conducted with rats running at 15 m/min for 2 min and then treadmill speed was increased every 2 min at a rate of 0.03 m/s (1.8–2 m/min) until the animals were not able to run (fatigue stage). The speed in the fatigue stage was estimated as 100% maximum oxygen consumption (VO2max). Lesser intensity was calculated as a percentage of speed in fatigue stage. For example, if animals were not able to run (fatigue stage) at the speed of 34 m/min, 60% VO2max = 60% × 34. At the end of each 2 weeks, the new speed according to the new estimated VO2max was calculated.

Each session consisted of 30 min of HIIT as shown in Table 1. The exercise program included three intensive intervals and low intensity. Intense periods were performed in 90 to 100% of estimated VO2max (speed in fatigue stage) for 4 min while low intensity periods was performed in 50 to 60% of estimated VO2max (speed in fatigue stage) for 2 min [20]. It should be noted that 50 to 60% of estimated VO2max (speed in fatigue stage) was considered for the warm-up and cool-down sessions. At this moment, a control group for the standardization of the effect of stress was placed on the treadmill for 15 min at a speed of 2 m/min. The training protocol continued for 5 days before sacrificing the rats.

**Table 1:** High-intensity interval training program

Steps	Warm-up	Three alternates		Cool-down
		High-intensity intermittent	Low-intensity intermittent	
Time (Minute)	6 Min	4 Min	2 Min	6 Min
Intensity (Vo2max)	50 to 60%	90 to 100%	50 to 60%	50 to 60%
First & second week (m/min)	18–22	32–36	18–22	18–22
Third & fourth week (m/min)	23–27	41–46	23–27	23–27
Fifth & sixth week (m/min)	27–32	48–54	27–32	27–32
Seventh & eighth week (m/min)	30–36	54–60	30–36	30–36
Ninth & tenth week (m/min)	35–41	62–69	35–41	35–41

### Sampling

Five days after completing the experimental stages of the research, rats were anesthetized using a combination of xylazine (3–5 mg/kg body weight) and ketamine (30–50 mg/kg body weight). Blood was collected in EDAT test tubes (anticoagulant) and immediately processed for plasma preparation during 10 min centrifugation at 1000×g. Plasma was frozen in liquid nitrogen and stored at − 70 C until they were taken to the laboratory.

### Evaluation of variables

Plasma levels of irisin, nesfatin-1 and resistin were measured by enzyme-linked immunosorbent assay (ELISA) method based on the manufacturer’s instructions. For irisin and nesfatin-1 measurement, the ELISA kit, (Bioassay technology Laboratory, China), with sensitivities of 0.03 and 16.23 ng/ml, respectively, was used. The plasma resistin level was measured by Biovendor kit (Biovendor Research and Diagnostic Products, Czech Republic) with a sensitivity of 0.25 ng/ml.

### Statistical analyses

Descriptive statistics were used to categorize and determine dispersion indices. The Kolmogorov–Smirnov test was used to determine data distribution. In order to estimate variations between the groups, the two-way Analysis of Variance (ANOVA) and Tukey Post hoc test were used. The repeated measure ANOVA was used to identify any difference in rats’ body mass for the duration of the study. Correlation was calculated with the Pearson Product Moment correlation. All significant levels were considered at *P* < 0.05 using the SPSS (Version 19) software.

## Results

**Fig. 1:**
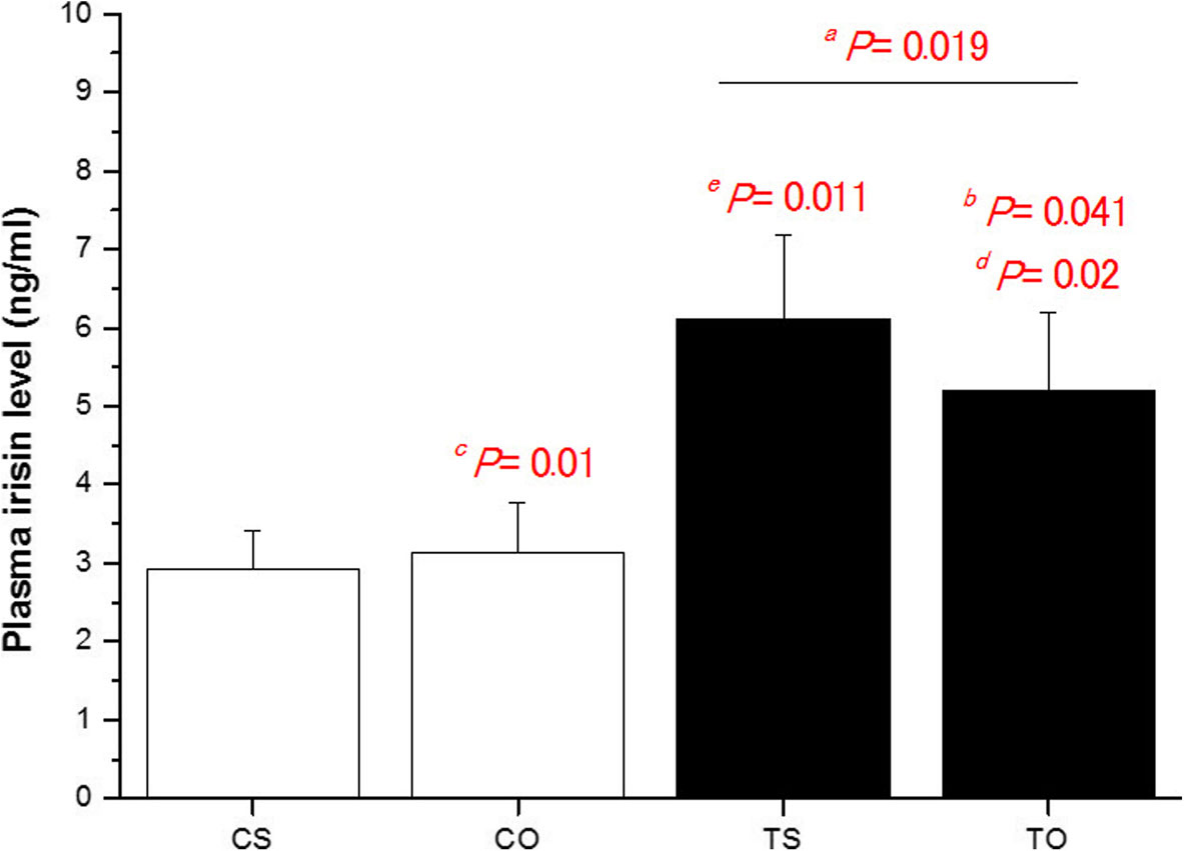
Data are express as mean ± standard deviation. CS: Control-Saline, TS: Training-Saline, CO: Control-FlaxOil supplement, and TO: Training-FlaxOil supplement. The rats are 10 in each group. ^*a*^ Training groups versus sedentary. ^*b*^ Interaction effects of exercise and supplement versus control groups. ^*c*^ CO versus CS. ^*d*^ CO versus TO. ^*e*^ TS versus CO

There was no difference in body mass (*P* = 0.376) (Table 2). Data analysis showed that there was a significant difference between the research groups regarding plasma irisin (*P* = 0.01). Using post hoc test showed that HIIT significantly increased plasma irisin levels compared to the control group (*P* = 0.019). There was also a significant difference between the control groups and the supplementary-HIIT (*P* = 0.041). Plasma irisin levels were higher in the TS group than CO group (*P* = 0.011). Plasma irisin levels were higher in the TO group than CO group (*P* = 0.02) (Fig. 1).

**Table 2:** Rat body mass (g). CS: Control-Saline, TS: Training-Saline, CO: Control-FlaxOil supplement, and TO: Training-FlaxOil supplement

Groups	Week									
	1	2	3	4	5	6	7	8	9	10
CS	207 ± 16.83	216.87 ± 15.79	226.62 ± 17.67	242.5 ± 15.09	266.87 ± 12.78	280.25 ± 16.20	296.87 ± 17.50	315 ± 16.68	331.25 ± 14.34	246.37 ± 16.69
TS	198.50 ± 18.17	207.12 ± 19.16	217.87 ± 20.93	235.62 ± 19.16	259.37 ± 16.86	267.75 ± 20.46	279 ± 22.09	292.37 ± 20.51	303.25 ± 19.41	317.75 ± 15.63
CO	204.50 ± 20.25	216.87 ± 17.17	223.62 ± 20.29	240.62 ± 19.97	267.12 ± 20.06	278.75 ± 19.85	292.12 ± 18.78	304.87 ± 19.5	324.62 ± 18.08	340 ± 17.92
TO	199.62 ± 12.01	205.50 ± 17.44	211.75 ± 15.82	232.62 ± 16.62	257.13 ± 18.78	267.12 ± 17.04	277.12 ± 17.16	301.12 ± 37.36	299 ± 16.63	321.87 ± 35.23

**Fig. 2:**
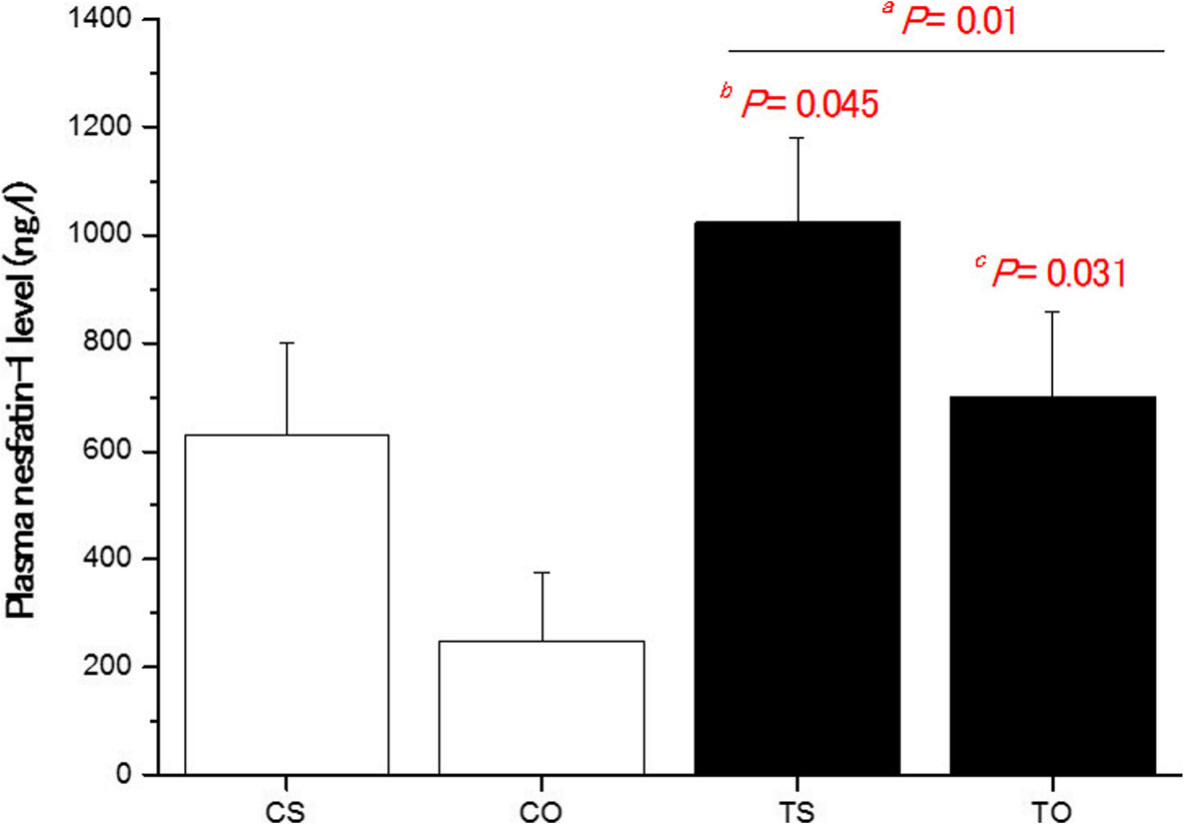
Data are express as mean ± standard deviation. CS: Control-Saline, TS: Training-Saline, CO: Control-FlaxOil supplement, and TO: Training-FlaxOil supplement. The rats are 10 in each group. ^*a*^ Training groups versus sedentary. ^*b*^ TS versus CO. ^*c*^ TO versus CO

In the case of nesfatin-1, data analysis showed a significant difference between the research groups (*P* = 0.01). Using post-test, there was a significant difference between the control and training groups (*P* = 0.01). HIIT significantly increased plasma nesfatin-1 levels compared to the control group (Fig. 2). Plasma nesfatin-1 was significantly higher in TS versus CO (*P* = 0.045) and TO versus CO (*P* = 0.031).

**Fig. 3:**
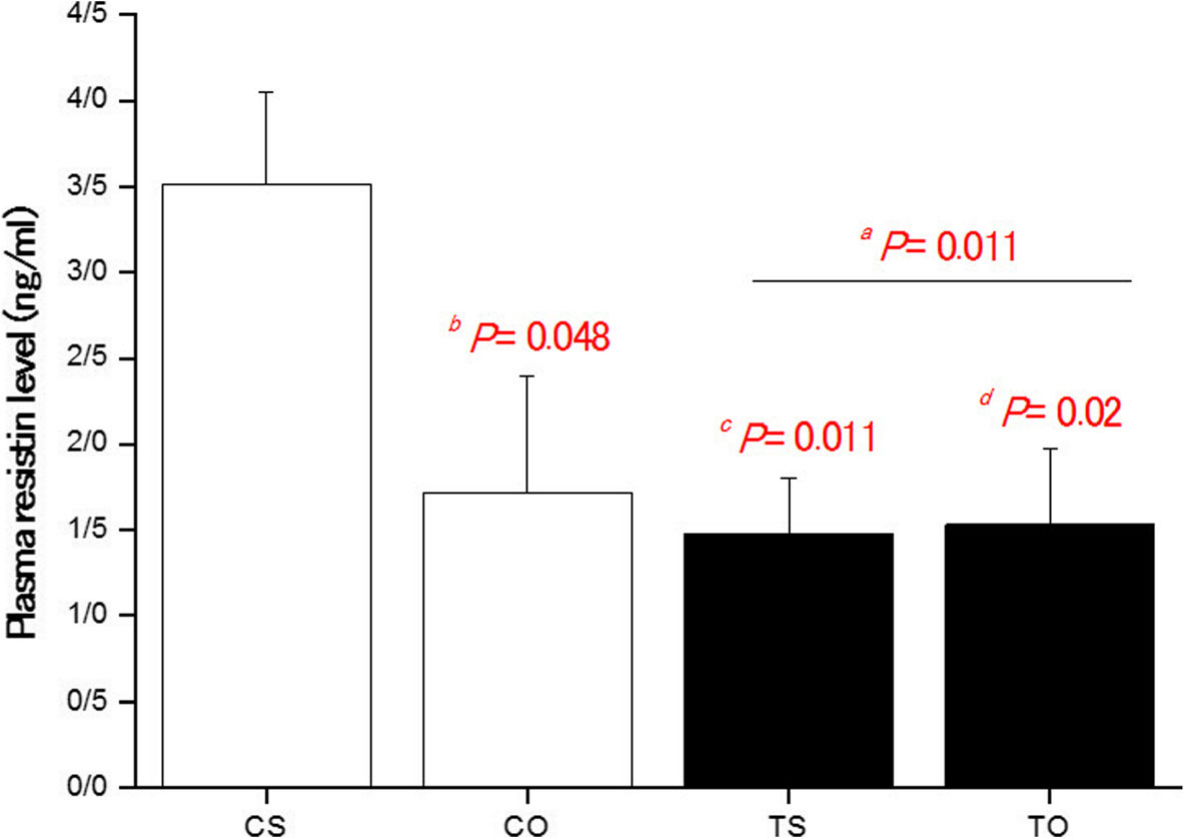
Data are express as mean ± standard deviation. CS: Control-Saline, TS: Training-Saline, CO: Control-FlaxOil supplement, and TO: Training-FlaxOil supplement. The rats are 10 in each group. ^*a*^ Training groups versus sedentary. ^*b*^ CO versus CS. ^*c*^ TS versus CS. ^*d*^ TO versus CS

Data analysis showed a significant difference between the research groups regarding resistin (*P* = 0.01). Using post hoc test showed a significant difference between CS group with CO (*P* = 0.048), TS (*P* = 0.011) and TO (*P* = 0.02). All groups showed lower plasma resistin levels than control (Fig. 3).

There was a positive and significant correlation between plasma levels of irisin and nesfatin-1 in TS (*P* = 0.04 and *r* = 0.64) and TO groups (*P* = 0.023 and *r* = 0.55) (Table 3). There was a negative and significant correlation between plasma irisin and resistin TS (*P* = 0.008 and *r* = − 0.62) and TO groups (*P* = 0.018 and *r* = − 0.53) (Table 3).

**Table 3:** Correlation between irisin levels in study groups. CS: Control-Saline, TS: Training-Saline, CO: Control-FlaxOil supplement, and TO: Training-FlaxOil supplement

Variables	Nesfatin-1		Resistin	
	*P*	*r*	*P*	*r*
CS	0.142	0.12	0.225	0.089
CO	0.211	0.104	0.064	- 0.23
TS	0.04 *	0.64	0.008 **	- 0.62
TO	0.023 *	0.55	0.018 **	- 0.53

* < 0.05; ** < 0.01

## Discussion

The most important findings of this study were 1- HIIT increased plasma levels of irisin, nesfatin-1 and decreased plasma levels of resistin; 2- Flaxseed oil reduced plasma levels of resistin; 3- The interaction between HIIT and flaxseed oil increased the plasma levels of irisin and decreased the plasma levels of resistin; 4- There is a positive and significant correlation between plasma levels of irisin and nesfatin-1; 5- There is a negative and significant correlation between plasma irisin and resistin.

The results of most previous studies indicated that physical training increased irisin. Daskalopoulou et al. [21] Showed that plasma levels of irisin increased in response to increased exercise load in active young people. In a study by Boström et al. [22], they showed that irisin increased after 3 weeks of aerobic training in rats and also resulted in an increase in energy expenditure and improved glucose levels. In addition, Huh et al. [23] showed that after 30 min of speed activity, irisin levels increased significantly. Research has shown that there are possible mechanisms to indicate how physical activity increases irisin levels. Researches have shown that physical activity increases Peroxisome proliferator-activated receptor gamma coactivator 1-alpha (PGC-1α) levels in skeletal muscle and the Fibronectin type III domain-containing protein 5 (FNDC5), the muscle-bearing membrane protein, that leads to irisin production [2]. AMP-activated protein kinase (AMPK’s) activation during HIIT is one of the factors that increases PGC-1α and irisin [24]. AMPK’s activation leads to the phosphorylation of PGC-1α as FNDC5’s modifier and irisin secretion [25]. Furthermore, PGC-1α activates peroxisome proliferator-activated receptor γ (PPARγ) which is involved in energy metabolism and stimulates FNDC5 and irisin increase [26]. It reported that there is a relationship between irisin levels and precursors of FNDC5 and PGC-1α [25]. The HIIT probably activates the PGC-1α activating signals, which also triggers a cascade of signals to change the phenotype of the adipose tissue. HIIT training causes energy consumption and heat production by increasing muscular tissue ratio to fat tissue and increasing UCP1 [24]. Thus, it triggers an increase in PGC-1α, FNDC5, and irisin [25]. Muscle production and secretion of irisin is also mediated by mothers against decapentaplegic homolog 3 (SMAD3), molecule that modulates energy metabolism and regulate body weight. SMAD3 suppresses FNDC5 and PGC-1α in skeletal muscle and negatively regulates plasma irisin [27]. Exercise induces phosphorylation of SMAD2 and subsequently, SMAD3 [27]. In recent years, studies have investigated the effect of physical exercises on nesfatin-1. Ghanbari-Niaki et al. evaluated the effect of 8 weeks of endurance training (5 days a week for 60 min at 25 m/min speed with zero gradient) on tissue nesfatin-1 gene expression and plasma levels of nesfatin-1 [28]. Their results showed that training increased the expression and nesfatin-1 plasma levels, which is related to plasma high-density lipoproteins (HDL) concentration. Nesfatin is involved in the regulation of blood glucose, improves insulin sensitivity, energy homeostasis, and metabolism [29]. The effect of exercise on nesfatin-1 has not been identified clearly and their effects after HIIT have not been studied. However, possible mechanisms exist. Studies have shown that nesfatin-1 is affected by various factors. For example, starvation in rats decreases serum nesfatin-1 levels by up to 18%. On the other hand, it has been reported that nesfatin-1 levels returned to normal levels, 1 to 12 h after refeeding [29]. In addition, some studies have shown that there is a direct relationship between nesfatin-1 and cortisol levels. In a study by [30], central injection of nesatin-1 increased adrenocorticotropin levels. According to previous studies, all of these factors are higher in the HIIT protocol, which can be considered as a possible cause for increased nesfatin-1 and as a result, these methods compared studies that did not indicate any changes. The adipose tissue also secretes various inflammatory cytokines that affect the expression and secretion of adipokines. Tumor necrosis factor-α (TNF-α) is one of these factors that has varying effects on adiponectin, leptin and nesfatin-1. Studies have shown that TNF-α, Interleukin 6 (IL-6) and insulin increase the intracellular expression of nesfatin-1 in fat-cultured cells [31]. These findings show that the expression and secretion of nesfatin-1 is regulated from different pathways. Some clinical studies have reported that there is a significant relationship between nesfatin-1 and insulin sensitivity [32]. Therefore, it is likely that physical activity, directly or indirectly alters the level of insulin and cortisol, as well as blood glucose. Physical activity also affects nesfatin-1 levels, and consequently, plays a role in improving insulin sensitivity. Although these factors were not investigated in this study, further studies are required in this regard. Concerning resistin, it has been reported that it increases because of obesity and significantly reduces by exercise and caloric restriction [12]. Kadoglou et al. [33] showed that 16 weeks of HIIT had beneficial effects on reducing serum resistin in obese and diabetic men and women. Bludasi et al. [34] showed that 12 months of HIIT reduced serum resistin in diabetic and obese people. Jamali et al. [35] showed that HIIT can reduce resistin gene expression in adipose tissues of obese rats as a pro-inflammatory agent. Thus far, the mechanism that explains the effect of HIIT on resistin has not been determined but possible explanations are available. A previous study showed that regular moderate-intensity physical training suppresses the expression of dual specificity protein phosphatase 1 (DUSP1), increases the expression of PGC-1α and reduces the activities of N-terminal kinases (JNK) and extracellular signal–regulated kinases (ERK) [36]. They concluded that the effects of anti-inflammatory exercise might be related to the supression of nicotinamide adenine dinucleotide phosphate (NADPH) oxidase, ERK1/2, and stress-activated protein kinases (SAPK)/JNK activities, and increases in superoxide dismutase 1 (SOD-1) gene expression. In our study, we reported decreased resistin levels after 10 weeks of HIIT of the beneficial effects of this type of training is similar to regular moderate continuous training.

Another finding of this study is the effect of flaxseed oil supplementation on the irisin and resistin levels. Flaxseed oil increases irisin levels. On the other hand, plasma resistin levels were decreased in response to flax seed oil. Thus far, a direct study has not investigated the effects of flaxseed oil on irisin and resistin, and there is no specific mechanism. It is well stablished that fruits, vegetables, and herbal plants are useful due to their components. For example, carotenoids (fat-soluble pigments) produced by plants and microorganisms and highly present fruits, vegetables, seaweeds and some seafood and a fundamental component of Mediterranean foods, are already known to decrease the incidence and prevalence of cardiovascular events, perhaps via their antioxidant action on free radicals or by acting as anti-inflammatory molecules [37, 38]. It has been shown that amelioration in calculated Framingham Risk Score in patients suffering from metabolic syndrome and undergoing nutraceutical administration [39]. Nutraceuticals (a food or part of a food that provides medical or health benefits, including the prevention and/or treatment of a disease [40]) are able to interact with several biochemical pathways in lipid metabolism [37]. Flaxseed oil contains a plant-based omega-3, alpha-linolenic acid (ALA), thus, the effects of flaxseed oil on irisin and resistin may related to ω-3. In a study by Ansari et al. [41], the use of omega-3 supplementation at 1250 mg three times daily increased irisin serum levels in diabetic patients. Vaughan et al. [42] showed that omega-3 consumption increased the expression of PGC-1α genes and skeletal muscle irisin. Tortosa-Caparros et al. [43] revealed that omega-3 and -6 consumption decreased cardiovascular disease by reducing resistin. Mostowik et al. [44] reported 11.3% decrease in resistin after omega-3 consumption which is associated with a reduction in coronary heart disease.

## Conclusion

In conclusion, the present study indicated the positive effect of HIIT and flaxseed oil in improving plasma irisin, nesfatin-1 and resistin levels. Probably, these changes are considered as mechanisms for increasing metabolism and reducing metabolic syndrome in patients, and thus require further research. In addition, further investigations into certain mechanisms related to the effects of HIIT and flaxseed oil on irisin, nesfatin-1, and resistin considering DUSP1, PGC-1α, JNK, ERK, NADPH oxidase, SOD-1, SMAD3, FNDC5, and UCPs are required.

## Abbreviations

AMPK: AMP-Activated Protein Kinase
CIDEA: Cell Death-Inducing DFFA-Like Effector A
CO: Control-FlaxOil supplement
CPT1B: Carnitine Palmitoyltransferase 1B
CS: Control-Saline
DIO2: Deiodinase, Iodothyronine Type 2
ERK: Extracellular signal–Regulated Kinases
FNDC5: Fibronectin Type III Domain-Containing Protein 5
HDL: High-Density Lipoproteins
HIIT: High-Intensity Interval Training
IL-6: Interleukin 6
JNK: N-terminal kinases
NADPH: Nicotinamide Adenine Dinucleotide Phosphate oxidase
NIH: National Institutes of Health
PGC-1α: Peroxisome Proliferator-Activated Receptor Gamma Coactivator 1-Alpha
PPARγ: Peroxisome Proliferator-Activated Receptor γ
SAPK: Stress-Activated Protein Kinases
SMAD3: Mothers Against Decapentaplegic Homolog 3
SOD-1: SuperOxide Dismutase 1
TNF-α: Tumor Necrosis Factor-α
TO: Training-FlaxOil supplement
TS: Training-Saline
UCP1: Uncoupling Protein 1
ω-3: Omega-3
